# The Specific Action of Quinine in Malaria

**Published:** 1897-09

**Authors:** 


### The Specific Action of Quinine in Malaria.

Dr. E. C. Register, editor of the *Charlotte Medical Journal*, read a paper with this title before the North Carolina Medical Society.

After many years of study, both clinical and microscopical, the doctor arrives at the following conclusion in reference to the specific action of quinine in the continued form of malarial fever. He says a malarial fever without complications, will subside after the plasmodia of malaria disappears from the blood; that we have in quinine the means to completely eradicate malarial poison from the body; that malarial fever occurring in a previously healthy subject, and in the central United States, if at once recognized and properly treated, never ends in death; that it is speedily curable, never continues, provided the nature of the disease be recognized and appropriate treatment employed.

Dr. Register has made microscopical examinations of the blood of several hundred patients suffering with remittent malarial fever, and has studied closely and thoroughly the crescentic and ring-shaped bodies which he says are the forms of the parasite which is responsible for the continued types of this fever, and he finds that the reason quinine does not always effect these irregular forms of the poison, is on account of the usual defects in its administration. He contends that the drug is very imperfectly absorbed when given by the stomach, and when the patient has a temperature of over 102 degrees. He says that in cases of continued malarial fever, if distinct and well marked intermissions of the fever are produced artificially by the use of antipyrine, antifebrine and phenacetine, the crescentic and ring-shaped bodies will disappear after the administration of quinine as quickly as the spherical bodies that are found in an ordinary case of intermittent fever. In reference to the belief that the forms of the parasite that inhabit the blood cells are not acted on by quinine, he says: "There is no doubt in my mind that this belief is not erroneous. Besides my own observations, I have been able to collect the opinions of thirty-two authors touching upon this point, and twenty-eight out of the thirty-two believe that the endo-globular or intra-corpuscular forms are not, on this account, the cause of an uncontrollable fever, and that its proximity to the blood cell does not, in any way, protect it from the action of quinine."—*St. Louis Medical Era.*

